# Multiomics analysis of cultured mouse periodontal ligament cell-derived extracellular matrix

**DOI:** 10.1038/s41598-023-51054-8

**Published:** 2024-01-03

**Authors:** Masaru Kaku, Lay Thant, Azusa Dobashi, Yoshiki Ono, Megumi Kitami, Masaru Mizukoshi, Moe Arai, Hajime Iwama, Kohei Kitami, Yoshito Kakihara, Masaki Matsumoto, Isao Saito, Katsumi Uoshima

**Affiliations:** 1 Division of Bio-Prosthodontics, Faculty of Dentistry and Graduate School of Medical and Dental Sciences, Niigata University, Niigata, Japan; 2 Division of Orthodontics, Faculty of Dentistry and Graduate School of Medical and Dental Sciences, Niigata University, Niigata, Japan; 3 Division of Dental Pharmacology, Faculty of Dentistry and Graduate School of Medical and Dental Sciences, Niigata University, Niigata, Japan; 4 Department of Omics and Systems Biology, Graduate School of Medical and Dental Sciences, Niigata University, Niigata, Japan; 5 Present Address: Division of Bio-Prosthodontics, Faculty of Dentistry and Graduate School of Medical and Dental Sciences, Niigata University, 2-5274, Gakkocho-dori, Chuo-ku, Niigata, Niigata 951-8514 Japan

**Keywords:** Gene expression, Biomimetics, Proteomics

## Abstract

A comprehensive understanding of the extracellular matrix (ECM) is essential for developing biomimetic ECM scaffolds for tissue regeneration. As the periodontal ligament cell (PDLC)-derived ECM has shown potential for periodontal tissue regeneration, it is vital to gain a deeper understanding of its comprehensive profile. Although the PDLC-derived ECM exhibits extracellular environment similar to that of periodontal ligament (PDL) tissue, details of its molecular composition are lacking. Thus, using a multiomics approach, we systematically analyzed cultured mouse PDLC-derived ECM and compared it to mouse PDL tissue as a reference. Proteomic analysis revealed that, compared to PDL tissue, the cultured PDLC-derived ECM had a lower proportion of fibrillar collagens with increased levels of glycoprotein, corresponding to an immature ECM status. The gene expression signature was maintained in cultured PDLCs and was similar to that in cells from PDL tissues, with additional characteristics representative of naturally occurring progenitor cells. A combination of proteomic and transcriptomic analyses revealed that the cultured mouse PDLC-derived ECM has multiple advantages in tissue regeneration, providing an extracellular environment that closely mimics the environment in the native PDL tissue. These findings provide valuable insights for understanding PDLC-derived ECM and should contribute to the development of biomimetic ECM scaffolds for reliable periodontal tissue regeneration.

## Introduction

The periodontal ligament (PDL) is a highly specialized fibrous tissue that anchors the tooth to the alveolar bone. The PDL maintains its thin non-mineralized layer despite existing between two mineralized tissues (i.e., the tooth root cementum and alveolar bone). The PDL is important for oral function, including the dissipation of masticatory force, tooth eruption, and neuronal feedback^1^. Various pathological conditions, such as periodontitis and occlusal trauma, irreversibly damage the PDL, resulting in tooth loss. Therefore, regeneration of the PDL is essential to restore the structure and function of the periodontium and prevent tooth loss.

The extracellular matrix (ECM) is a principal tissue component that plays a pivotal role in tissue function. It primarily provides a structural framework for tissues whose mechanical properties rely on tissue-specific ECM composition and their secondary structures^2,3^. Another important function of the ECM is to provide mechanical and biochemical cues to cells as an extracellular microenvironment^4^. Because the ECM plays a key role in regulating cell behavior, and the composition and organization of the ECM vary depending on the tissue type, it is important to understand the tissue-specific ECM profile in the context of tissue regeneration.

The major ECM components of the PDL are fibrillar collagens (e.g., type I and III), minor collagens (e.g., types V, VI, and XII), proteoglycans, and ECM glycoproteins^1^. We recently reported the comprehensive ECM profile of mouse molar PDL using laser microdissection and mass spectrometry-based proteomic analysis with ECM-oriented data curation^5^. Our data revealed that type I collagen accounted for 86.5% and types III and XII collagens each accounted for 4.5% of the total collagen. The most abundant proteoglycan/ECM glycoprotein is periostin (POSTN), followed by lumican (LUM), asporin (ASPN), biglycan (BGN), and tenascin-N (TNN)^5^. Notably, the ECM proportion, together with the morphological characteristics of collagen fibers, was significantly changed by mechanical stress induced by an orthodontic appliance^5^. These data are in accordance with those of previous proteomic analyses showing dynamic changes in the ECM profile under different physiological environments^6,7^. Hence, altered ECM proportions in different physiological states may modulate cell behavior, playing essential roles in the homeostasis and regeneration of the periodontal tissue.

Recent advances in tissue engineering offer various approaches for the regeneration of the PDL^8^. One approach is the use of periodontal ligament cell (PDLC)-derived ECM, which is obtained through the decellularization of cultured PDLCs. The PDLC-derived ECM maintains its structural integrity, preserves growth factors, and supports the repopulation of allogeneic cells^9^. It was found that culturing PDLCs on PDLC-derived ECM maintained their stemness and osteogenic potential better than culturing on tissue culture polystyrene^10^. This method also induced osteoblastic differentiation of allogeneic MSCs^11^ and led to the differentiation of induced pluripotent cells into PDL stem cell-like cells^12^. Because of such cell instructive features, the PDLC-derived ECM is considered to preserve the original ECM composition of native PDL tissue to some extent. However the detailed profile of the PDLC-derived ECM remains elusive. Therefore, in the present study, we aimed to systematically analyze the PDLC-derived ECM and compare it with that in the native PDL tissue in mice. To gain a comprehensive and detailed understanding, we employed a multiomics approach, integrating data from different omics technologies (namely proteomics and transcriptomics).

## Results

### Proteomic characterization of cultured mouse PDLC-derived ECM

**Figure 1:**
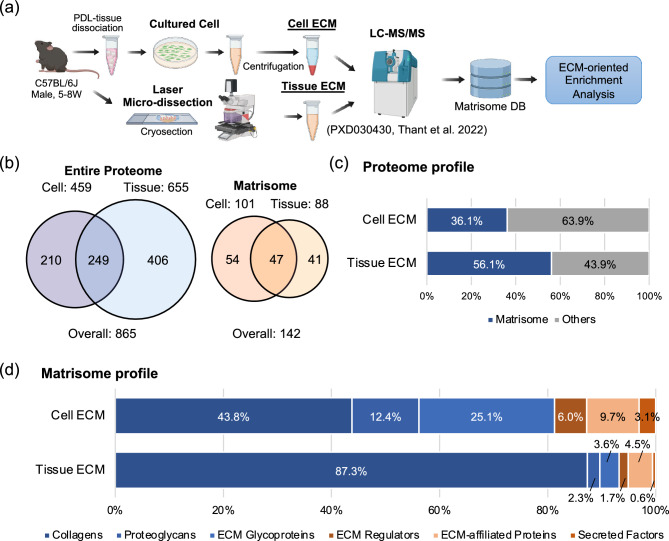
Matrisome protein profiles of cultured mouse PDLCs and PDL tissue. (**a**) Experimental setting of the proteomic analysis. (**b**) Number of proteins detected in the ECM-rich fraction of cultured PDLCs (Cell) and native PDL tissue (Tissue). The number of detected proteins in the entire proteome and that in matrisome is shown separately. (**c**) Occupancy of matrisome proteins in the Cell and Tissue ECM within the entire proteome. (**d**) Matrisome protein profiles of the Cell and Tissue ECM. Occupancy was calculated based on the exponentially modified protein abundance index (emPAI) value of each matrisome subclass divided by the sum emPAI value of total matrisome proteins.

To elucidate the proteomic profile of the PDLC-derived ECM, we conducted an ECM-oriented proteomic analysis on cultured PDLCs (Fig. 1a). The ECM-enriched fraction of cultured PDLCs (Cell ECM) was prepared by decellularization and analyzed using liquid chromatography with tandem mass spectrometry. ECM-oriented data curation was performed using the Matrisome database^13^. Previously reported proteomic data of mouse PDL tissue dissected using laser microdissection^5^ were reanalyzed and used for the Tissue ECM. The number of detected proteins in Cell ECM was 459, while that in Tissue ECM was 655 (Fig. 1b). The number of matrisome proteins in Cell and Tissue ECM was 101 and 88, respectively. Forty-seven out of 142 matrisome proteins in total were detected in both Cell and Tissue ECM. Matrisome proteins accounted for 36.1% of the entire proteome in Cell ECM and for 56.1% of that in Tissue ECM (Fig. 1c). The relative proportions of the matrisome subclasses, collagens, proteoglycans, and ECM glycoproteins in Cell ECM were 43.8%, 12.4%, and 25.1%, respectively (Fig. 1d). Compared to Tissue ECM, the proportion of collagens was significantly lower in Cell ECM, while that of other matrisome subclasses (i.e., proteoglycans, ECM glycoproteins, ECM regulators, ECM-affiliated proteins, and secreted factors) was significantly higher (Fig. S1).

**Figure 2:**
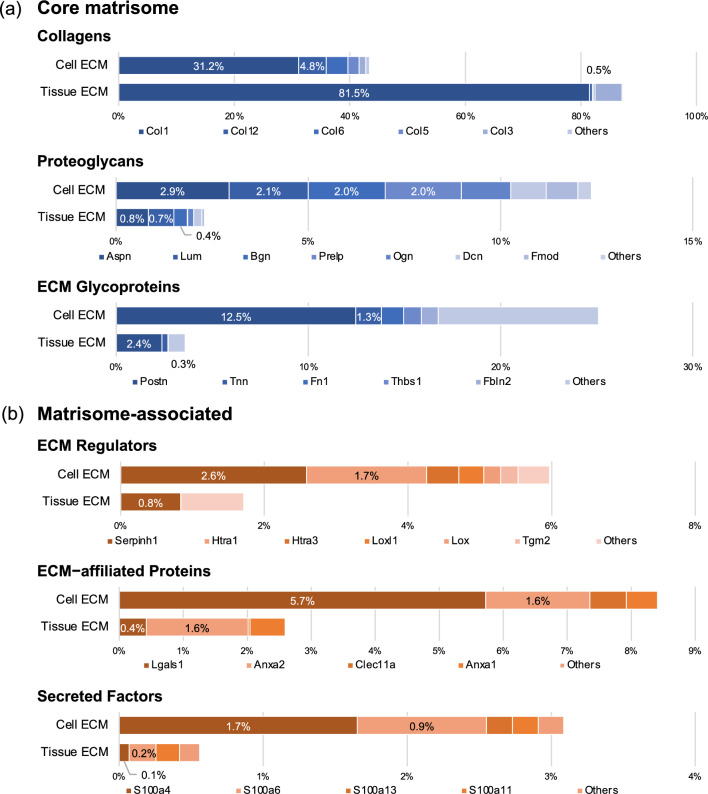
Protein profile of matrisome subclasses in cultured mouse PDLCs and PDL tissue. Detailed profiling of the (**a**) core matrisome and (**b**) matrisome-associated proteins in the Cell and Tissue ECM. Occupancy was calculated based on the emPAI value of each matrisome protein divided by the sum emPAI value of total matrisome proteins. Protein order in each matrisome subclass corresponds to the descending order of emPAI value in the Cell ECM.

The detailed protein proportion in each matrisome subclass showed that the most abundant collagen in Cell ECM was type I collagen, occupying 31.2% of the matrisome, while that in Tissue ECM was 81.5% (Fig. 2a). The second major collagen in Cell ECM was type XII, followed by types VI, V, and III (Fig. S2). The major proteoglycans in Cell ECM were ASPN, LUM, BGN, and prolargin (PRELP), each accounting for 2%–3% of the matrisome. The most abundant ECM glycoprotein in Cell ECM was POSTN, which accounted for 12.5% of the matrisome and ranked second in abundance in Cell ECM matrisome.

The matrisome-associated proteins were also more abundant in Cell ECM compared to Tissue ECM (Figs. 2b, S3). HSP47 (Seripnh1) is the major ECM regulator in both Cell and Tissue ECM. The high-temperature requirement (Htr) family of serine proteases (HTRA1 and HTRA3) and collagen-modifying enzymes (LOX, LOXL1, and LOXL2) were detected in Cell ECM, but not in Tissue ECM. A noticeable amount of galectin-1 (LGALS1) was detected in Cell ECM, accounting for 5.7% of the matrisome. Annexin-family proteins (ANXA1 and ANXA2), Ca^2+^ phospholipid-binding proteins, were detected in both Cell and Tissue ECM with similar occupancy. The S100 family (S100A4, S100A6, S100A13, and S100A11) of secreted factors was detected both in the Cell and Tissue ECM, with the Cell ECM showing a higher proportion of these factors.

### Overrepresentation analysis using the differentially expressed proteins (DEPs) in cultured mouse PDLC-derived ECM

**Figure 3:**
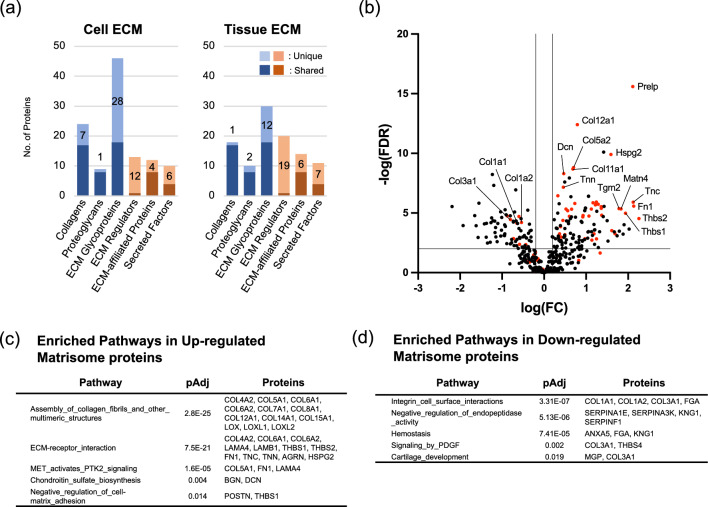
Enrichment analysis of differentially expressed matrisome proteins in cultured mouse PDLCs and PDL tissue. (**a**) Number of detected proteins in matrisome subclasses in Cell and Tissue ECM. Matrisome proteins detected in both the Cell and Tissue ECM, and those only detected in either group are separately shown as Shared and Unique, respectively. Volcano plot of the differentially expressed proteins (DEPs) in the Cell ECM vs. those in the Tissue ECM. The matrisome proteins are shown in red dots. Top enrichment network of the overrepresentation analysis of (**c**) upregulated matrisome proteins and (**d**) downregulated matrisome proteins. FDR; false discovery rate, FC; fold change, pAdj; adjusted *p* value.

The number of collagens and proteoglycans in the detected proteins in Cell and Tissue ECM was similar, while the number of unique ECM glycoproteins was two-fold higher in Cell ECM than that in Tissue ECM (Fig. 3a). Many of the identified matrisome-associated proteins, namely ECM regulators, ECM-affiliated proteins, and secreted factors, were unique in either Cell or Tissue ECM.

DEPs were identified based on the proportion of individual proteins within a defined threshold (Fig. 3b). Many of the upregulated DEPs were matrisome proteins, whereas downregulated DEPs contained the most abundant collagen types in Tissue ECM: type I and III collagens. Overrepresentation analysis showed that the assembly of collagen fibrils and other multimeric structures (R-MMU-2022090, pAdj = 2.8E−25) and ECM-receptor interaction (mmu04512, pAdj = 7.5E−21) were the most enriched terms for upregulated DEPs (Fig. 3c). Integrin cell–surface interactions (R-MMU-216083, pAdj = 3.3E−07) showed the highest enrichment score for downregulated DEPs (Fig. 3d).

### Gene expression profiling of mouse PDLCs

**Figure 4:**
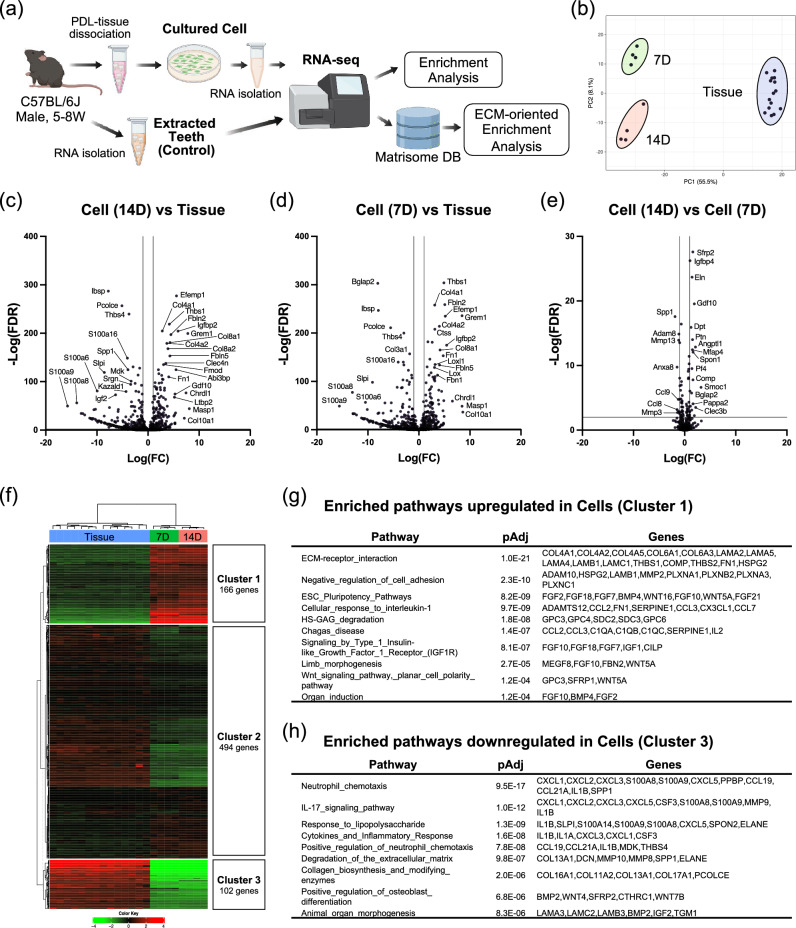
Gene expression profiling of cultured mouse PDLCs using native mouse PDL tissue as reference. (**a**) Experimental workflow of the comprehensive gene expression analysis. (**b**) Principal component analysis showing that the groups were significantly different from one another. Volcano plot of the differentially expressed genes (DEGs) in the (**c**) Cell (14D) vs. Tissue, (**d**) Cell (7D) vs. Tissue, (**e**) Cell (14D) vs. Cell (7D). (**f**) Heatmap showing k-means clustering of differentially expressed matrisome genes. Expression values of each biological replicate are indicated according to a color gradient. Cluster 1 represents 166 upregulated matrisome genes on cultured PDLCs compared to native PDL tissue. Cluster 3 represents 102 downregulated matrisome genes on cultured PDLCs. Top enrichment network of the overrepresentation analysis on (**g**) upregulated matrisome genes and (**h**) downregulated matrisome genes. FDR; false discovery rate, FC; fold change, pAdj; adjusted *p* value.

Cells under culture are affected by an extracellular environment composed of their own secreted ECM in the same way that cells in tissues are affected by their native ECM as an extracellular environment. To this end, the gene expression of cultured cells reflects the effect of their secreted ECM on the cells. To investigate the gene expression profile of cultured mouse PDLCs, total RNA was isolated from the cells after 7D and 14D of culture. Total RNA directly prepared from the PDL tissue of the extracted teeth was used as a control. Gene expression in cultured PDLCs and in cells of the PDL tissue was analyzed using RNA sequencing (RNA-seq), and subsequently pathway and process enrichment analyses were performed. A schematic representation of the experimental workflow is shown in Fig. 4a. Principal component analysis showed that the samples were significantly different from one another, with the 7D and 14D PDLCs showing a close association (Fig. 4b). Using k-means clustering with the top 2000 significant genes, we showed that 571 genes were upregulated and 1404 were downregulated in cultured PDLCs compared with that in PDL tissue cells (Fig. S4a). Gene ontology (GO) analysis of differentially expressed genes (DEGs) showed that the upregulated genes in cultured PDLCs shared enrichment terms related to the ECM (Fig. S4b, d) and the extracellular space (Fig. S4c). On the contrary, the most enriched terms for the downregulated genes were keratinization (Fig. S4b) and ribosomal genes (Fig. S4c, d).

### Network enrichment analysis using differentially expressed matrisome genes of cultured mouse PDLCs

GO analysis strongly indicated that the major difference between cultured PDLCs and PDL tissue at the transcriptomic level was the ECM status; therefore, we focused on the ECM and its regulatory genes (matrisome genes)^13^ to gain a deeper understanding of these differences. Differentially expressed matrisome genes were identified between the groups and are shown in the volcano plot (Fig. 4c, d). Significant differences in gene expression patterns were observed between the 14D PDLCs and PDL tissue samples (Fig. 4c) and between the 7D PDLCs and PDL tissue samples (Fig. 4d), whereas the difference between the 7D and 14D PDLCs was minor (Fig. 4e). Among the matrisome genes identified (a total of 997 genes), upregulated genes (Cluster 1; 166 genes) and downregulated genes (Cluster 3; 102 genes) were identified using k-means clustering (Fig. 4f). Overrepresentation analysis revealed that the ECM–receptor interaction (mmu04512, pAdj = 1.0E−21) was most significantly enriched for upregulated matrisome genes (Fig. 4g). The ESC Pluripotency Pathways (WP339, pAdj = 8.2E−09), mainly controlled by the FGF and Wnt signaling pathways, were identified as the third most enriched pathway in upregulated matrisome genes. Neutrophil chemotaxis (GO:0030593, pAdj = 9.5E−17) was the most significantly enriched pathway in the downregulated genes (Fig. 4h). Notably, the IL-17 signaling pathway (mmu04657, pAdj = 1.0E−12) as well as cytokines and inflammatory response (WP222, pAdj = 1.6E−08), both related to the immunological response, did not exist under the culture conditions and were enriched in downregulated matrisome genes. In addition, the positive regulation of osteoblast differentiation (GO:0045669, pAdj = 6.8E−06) was enriched for downregulated genes.

### Multiomic profiling of ECMs in cultured mouse PDLCs

**Figure 5:**
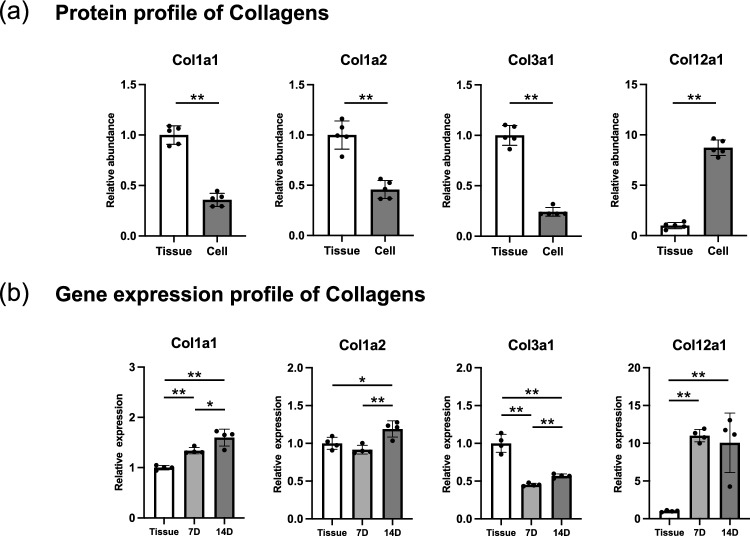
Protein and gene expression of collagens in cultured mouse PDLCs and PDL tissue. (**a**) Relative protein abundance of collagens in the Cell ECM compared with that in the Tissue ECM. (**b**) Relative gene expression of collagens in cultured PDLCs compared with that in cells from native PDL tissues. 7D, cultured PDLCs on day 7; 14D, cultured PDLCs on day 14. *p < 0.05, **p < 0.01.

**Figure 6:**
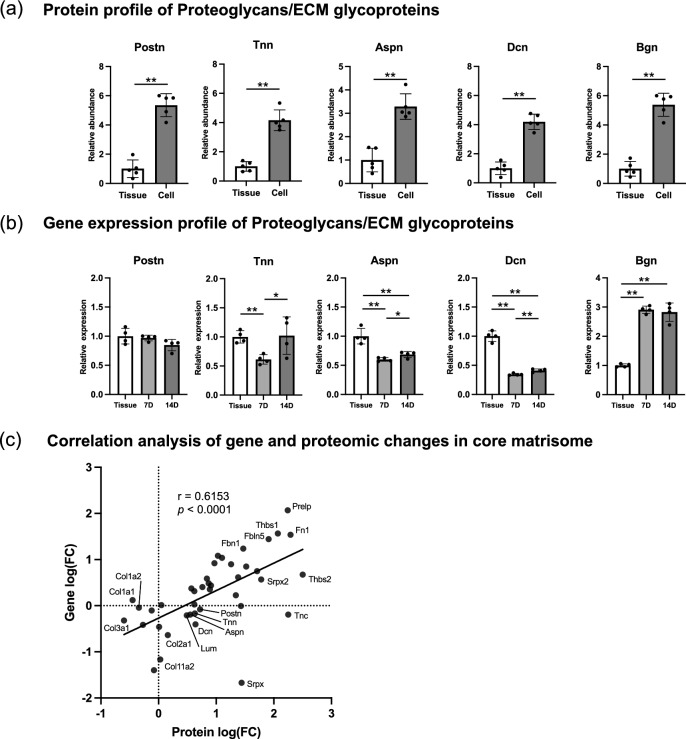
Protein and gene expression of proteoglycans/ECM glycoproteins in cultured mouse PDLCs and PDL tissue. (**a**) Relative protein abundance of proteoglycans/ECM glycoproteins in the Cell ECM compared with that in the Tissue ECM. (**b**) Relative gene expression of proteoglycans/ECM glycoproteins in cultured PDLCs compared with that in cells from native PDL tissues. (**c**) Correlation analysis of gene and proteomic changes of the core matrisome in cultured PDLCs. 7D, cultured PDLCs on day 7; 14D, cultured PDLCs on day 14. *p < 0.05, **p < 0.01.

To further analyze the details of ECMs and their regulators in cultured PDLCs, protein and gene expression changes were comparably analyzed (Figs. 5 and 6). The protein proportion of type I collagen significantly decreased (Fig. 5a), while its gene expression (*Col1a1* and *Col1a2*) increased (Fig. 5b). Both the protein proportion and gene expression of type III collagen (*Col3a1*) decreased, while those of type XII collagen increased. The proportion of major proteoglycans and ECM glycoproteins significantly increased (Fig. 6a). In contrast, their gene expression levels showed an unstable pattern (Fig. 6b). The expression of *Postn*, the gene encoding the second most abundant protein in the Cell ECM, did not change significantly. The expression of *Aspn* and *Dcn* decreased, while that of *Bgn* increased. Correlation analysis showed a significant correlation between changes in gene expression and the protein proportion of the core matrisome (Fig. 6c). Discrepancies in gene expression and protein abundance were observed mainly in high-abundance proteins in the Cell and Tissue ECM, such as COL1A1, POSTN, TNN, ASPN, and LUM.

Post-translational modifications of collagen are essential for the integrity of the ECM of PDL tissue^14^. We observed an increased abundance of collagen-modifying enzymes (LOX, LOXL, and LOXL2) in Cell ECM compared with that in Tissue ECM (Fig. 3b). To gain further insights into the secretion and maturation of collagen in cultured PDLCs, we analyzed the gene expression of collagen-modifying enzymes known to play essential roles in collagen biosynthesis^15^ (Fig. S5). All Lox family proteins (encoded by *Lox*, *Loxl1-4*) showed significantly higher gene expression in cultured PDLCs than that in PDL tissue. Gene expression of all lysyl hydroxylases (encoded by *Plod1-3*) and essential binding partners for lysyl hydroxylase 2 (*Plod2*), HSP47 (*Serpinh1*) and Fkbp65 (*Fkbp10*), increased in the cultured PDLCs. Among the prolyl 4-hydroxylases (*P4ha1-3*, *P4hb*), the gene expression of *P4ha1*, *P4ha3*, and *P4hb* was increased, while that of *P4ha2,* the predominant form in chondrocytes, osteoblasts, and endothelial cells^16^, was unchanged.

**Figure 7:**
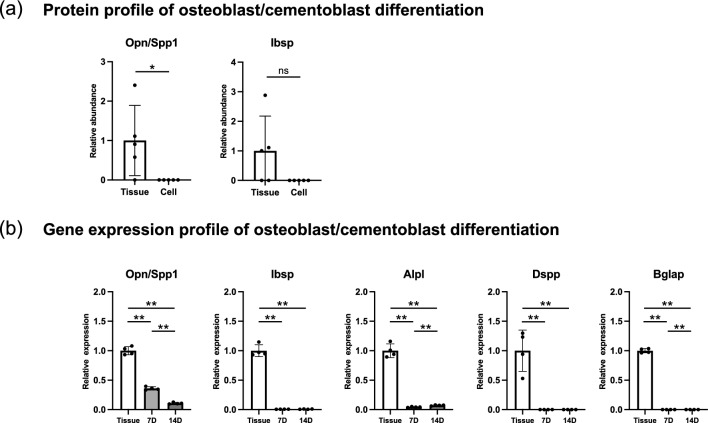
Protein and gene expression of osteoblast/cementoblast differentiation-associated components in mouse PDLCs and PDL tissue. (**a**) Relative abundance of osteoblast/cementoblast differentiation-associated proteins in the Cell ECM compared with that in the Tissue ECM. (**b**) Relative expression of osteoblast/cementoblast differentiation-associated genes in cultured PDLCs compared with that in cells from native PDL tissues. 7D, cultured PDLCs on day 7; 14D, cultured PDLCs on day 14. *p < 0.05, **p < 0.01.

Overrepresentation analysis indicated diminished osteoblastic differentiation of PDLCs in culture (Fig. 4h). We analyzed proteomic and gene expression changes in osteoblast/cementoblast-associated ECM (Fig. 7). The abundance of osteopontin (OPN/*Spp1*) and bone sialoprotein (BSP/*Ibsp*) tended to decrease (Fig. 7a). The large SD values of OPN and BSP may be due to the contamination of bone/cementum tissue during the laser microdissection procedure. The expression of *Spp1* and *Ibsp* was significantly decreased in cultured PDLCs (Fig. 7b), while that of other osteoblast/cementoblast differentiation markers, including *Alpl*, *Dspp*, and *Bglap*, was decreased.

### Gene expression profiling of cultured mouse PDLCs

**Figure 8:**
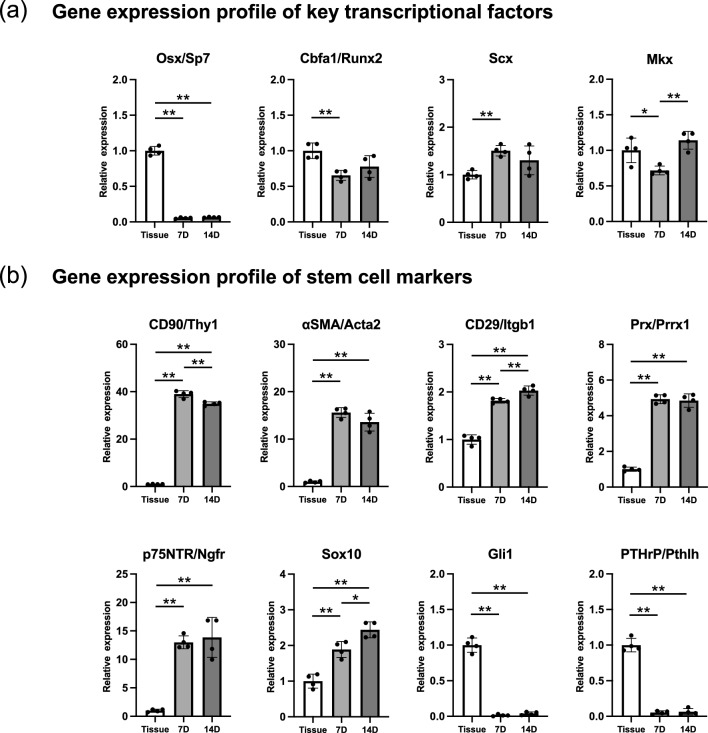
Gene expression of key transcriptional factors and stem cell markers in cultured mouse PDLCs and PDL tissue. Relative gene expression of (**a**) key transcriptional factors and (**b**) stem cell markers in cultured PDLCs compared with that in cells from native PDL tissue. 7D, cultured PDLCs on day 7; 14D, cultured PDLCs on day 14. *p < 0.05, **p < 0.01.

Gene expression of transcription factors critical for osteoblast/cementoblast differentiation, osterix (*Sp7*) and Cbfa1/Runx2 (*Runx2*), and important for PDL homeostasis, scleraxis (*Scx*) and mohawk (*Mkx*), was analyzed (Fig. 8a). The gene expression of *Sp7* and *Runx2* significantly decreased, consistent with that of osteoblast/cementoblast markers (Fig. 7b). Gene expression of *Scx* increased in cultured PDLCs whereas that of *Mkx* decreased on day 7 but increased on day 14.

Overrepresentation analysis generated the enrichment term ESC Pluripotency Pathways (WP339, pAdj = 8.2E−09), indicating enhanced stem cell properties or an increased proportion of stem cells in cultured PDLCs (Fig. 4g). We selected stem cell marker genes, which are reported to have stem cell-like characteristics in PDL tissue (Fig. 8b). Gene expression of MSC markers CD90 (*Thy1*), αSMA (*Acta2*), and CD29 (*Itgb1*), and neural crest cell markers p75NTR (*Ngfr)* and *Sox10,* significantly increased in cultured PDLCs. In contrast, the gene expression of *Gli1*, a Wnt signal responsive gene, and PTHrP (*Pthlh*), confirmed to play crucial roles in tooth root development, was significantly decreased in cultured PDLCs.

## Discussion

The ECM is a complex network of proteins that provides mechanical and biochemical signaling cues to guide cell behavior^4^. The composition and organization of the ECM vary depending on the tissue and environment; therefore, understanding these differences is important in the context of tissue regeneration. Here, we systemically analyzed the PDLC-derived ECM using a multiomics approach. The major change in the matrisome protein profile of the cultured PDLCs was the lower proportion of fibrillar collagens (type I and III collagens) compared to that in PDL tissue. Fibrillar collagens play a key role in tissue integrity and the tensile behavior of the PDL, whereas the rest of the ECM components, such as proteoglycans and ECM glycoproteins, are responsible for the viscoelastic response to compressive force^17^. Therefore, a lower proportion of fibrillar collagens with an increased proportion of glycoproteins results in significant changes in mechanical properties (e.g., tensile strength and elastic modulus). This also affects cell behavior, as the ECM provides an extracellular microenvironment to the surrounding cells^18^. We recently reported changes in the matrisome protein profile of the mouse PDL in response to mechanical stress^5^. Similar to the cultured PDLC-derived ECM, decreased levels of fibrillar collagens with increased glycoprotein levels have been observed during orthodontic tooth movement, in which mechanical stress-induced tissue remodeling actively takes place. Furthermore, the levels of matrisome proteins that are preferentially expressed in the PDL, such as POSTN^19^, TNN^20^, ASPN^21^, and S100A4^22^, tended to increase in the ECM of cultured PDLCs. Our results indicate that the cultured PDLC-derived ECM preserves PDL-specific proteins and has characteristics similar to those of PDL tissue with active collagen fibrogenesis.

Previous studies have shown differences between the gene expression profiles of PDLCs in culture and in native PDL tissues. The cells under culture are influenced by their secreted ECM, and as a result, the gene expression in cultured PDLCs may partially represent the impact of their own-secreted ECM on the cells. In a recent study comparing the gene expression profiles of cultured PDLCs and cells in the PDL tissue of extracted human teeth using cap analysis of gene expression, the expression of many ECM genes, including *Postn*, *Aspn*, osteomodulin (*Omd*), *Tnn*, *Spp1*, and osteocalcin (*Ocn*), was remarkably decreased in cultured PDLCs^23^. Another study comparing human PDL tissue and cultured PDLCs using microarray reported that *Alpl*, *Ibsp*, *Postn*, *Fmod*, *Spp1*, and *Omd* were highly expressed in the PDL tissue, whereas gremlin (*Grem1*), follistatin (*Fst*), *CTGF,* and *FGF5* were expressed at higher levels in cultured PDLCs^24^. Subtractive hybridization analysis revealed that type I and III collagen, *Lum*, *Postn*, *Aspn*, and follicular dendritic cell-secreted protein (*Fdcsp*) were highly expressed in the PDL tissue compared with that in cultured PDLCs^25^. Our gene expression data obtained using RNA-seq are consistent with those of previous studies showing that the expression of genes encoding ECM glycoproteins (*Postn* and *Tnn*), proteoglycans (*Aspn* and *Lum*), and osteoblast/cementoblast differentiation proteins (*Alpl*, *Ibsp*, *Spp1*, and *Ocn*) decreased in the cultured PDLCs compared with that in the PDL tissue. Moreover, our comprehensive gene expression analysis revealed that the major transcriptomic changes in cultured PDLCs were associated with the ECM. It remains unclear as to which cell population is responsible for the ECM turnover in the PDL tissue because the cells comprising the PDL tissue are heterogeneous and not well characterized^1,26^. The difference in the cell population between culture and in the native tissue also needs to be taken into consideration.

Type I collagen in the PDL undergoes distinct post-translational modifications that markedly affect the formation of intermolecular cross-links and determine the structural and mechanical properties of the tissue^14,15^. In the present study, collagen-modifying enzymes, such as LOX, LOXL1, and LOXL2 were detected only in the proteome datasets of the Cell ECM but not in the Tissue ECM (Fig. S3). In addition, the gene expression of collagen-modifying enzymes tended to increase in cultured PDLCs compared with that in the PDL tissue, which is indicative of activated collagen fibrogenesis (Fig. S5). We previously reported that mechanical stress accelerates the maturation of collagen cross-linking through post-translational modifications of type I collagen in human PDLCs^27^. Because cultured PDLCs are free from occlusal loading, some biomechanical cues that are crucial for ECM organization and maturation may be lacking. Although collagen-modifying enzymes are increased in cultured PDLCs, fibrillar collagens are most likely less cross-linked in the cultured PDLCs than in the PDL tissue, owing to the absence of occlusal loading. Furthermore, an enrichment pathway of ECM–receptor interactions in upregulated DEPs indicated accelerated ECM turnover because cell–ECM interactions generally stimulate ECM degradation^28^. Increased expression of MMP-2, a gelatinase with a wide substrate range, was observed in cultured PDLCs at both the mRNA and protein levels. In accordance with the matrisome profiles, the ECM of cultured PDLCs has an immature status with increased turnover.

Among the well-characterized transcriptional factors in PDL, the gene expression of *Cbfa1/Runx2*^29^, *Scx*^30^, and *Mkx*^31^ was well maintained, while that of *Sp7*^32,33^ was significantly decreased in cultured PDLCs. It has been shown that the deletion of *Sp7* in *Col1-* or *Ocn*-expressing cells in mice results in a short root with thin root dentin^33^, while the conditional deletion of *Cbfa1/Runx2* in *Gli1*-lineage cells completely inhibits tooth root formation^29^. Despite a slight difference in the Cre-driver system, deletion of *Cbfa1/Runx2* resulted in a more severe phenotype compared to *Sp7*, indicating that *Cbfa1/Runx2* is essential for tooth root development and also most likely for its maintenance. Therefore, the decreased expression of *Sp7* in the cultured PDLCs may not significantly affect PDLC characteristics. Notably, the gene expression of *Mkx* and *Scx* showed the opposite pattern in 7D PDLCs; the expression of *Scx* increased, while that of *Mkx* decreased. This observation is consistent with a recent study showing the mutually exclusive role of *Scx* and *Mkx* in PDL, as revealed using the single-cell RNA-seq of *Mkx*-KO mice^34^.

As the PDL is abundant in tissue stem cells, and they contribute substantially to periodontal tissue regeneration, the identification and characterization of the tissue stem cells in the PDL has attracted considerable research interest^35^. Cultured PDLCs express several stem cell markers, including CD29/*Itgb1*, CD90/*Thy1*, and Stro-1^36^. In addition, lineage-tracing studies have shown that αSMA/*Acta2*- ^37^, *Axin2*- ^38,39^, *Prrx1*- ^40,41^, *Gli1*- ^42^, PTHrP/*Pthlh*^43^*,* and CD90/*Thy1*-expressing cells^39^ contribute to PDL tissue maintenance. Furthermore, cultured PDLCs had approximately 50-fold higher Stro-1 protein levels than cells in PDL tissue^24^, and all the expanded cells isolated from the molar surface were *Gli1*-lineage cells^42^. Therefore, cultured PDLCs represent a naïve cell population; possibly, they are naturally occurring progenitor cells^24^. In our experimental settings, the cultured PDLCs were affected by the ECM produced by them. Consistent with the previous notion, our pathway enrichment analysis indicated that the ESC pluripotency pathway (WP339), mainly controlled by FGF and Wnt signaling, was enhanced in cultured PDLCs. Gene expression data also indicated a significant increase in MSC markers (CD90/*Thy1*, aSMA/*Acta2*, CD29/*Itgb1*, and *Prrx1*) and neural crest cell markers (p75NTR/*Ngfr* and *Sox10*). In contrast, the expression of genes expressed by well-characterized stem cell-like populations in PDL, *Gli1*, and PTHrP/*Pthlh,* significantly decreased. Our results partially support the notion that cultured PDLCs represent naturally occurring progenitor cells^24^, capable of accelerating tissue regeneration. Collectively, the PDLC-derived ECM possibly guides the cells toward a tissue progenitor cell state.

Currently, label-free proteomics is a versatile protein-quantification method in bottom-up proteomics^44^. Label-free spectral counting-based protein quantification relies on the abundance of tryptic peptides in each sample. Because larger proteins generate more peptides in theory, the spectral counts of these proteins tend to be overrepresented. In the present study, we used exponentially modified protein abundance index (emPAI), a spectral counting-based relative protein quantification technique, in which the number of observed peptides was divided by the number of theoretically observable peptides per protein^45^. We previously reported the matrisome profile of mouse PDL tissue using another spectral counting-based quantification technique, normalized spectral abundance factor (NSAF), which normalized the spectral counts with respect to the protein length^46^. Despite the same proteome data set being used, the collagen proportion of the Tissue ECM matrisome was 43.8% in the current study, but it was 55 to 58% in the previous report using NSAF^5^. Although it is difficult to ascertain the algorithm that is most suitable for evaluating the matrisome profile, it should be noted that the difference in the normalization algorithm affects relative protein quantification in spectral counting-based protein quantification.

To the best of our knowledge, our study is the first to show a comprehensive profile of cultured mouse PDLC-derived ECM in reference to the mouse PDL tissue. A combination of proteomic and transcriptomic analyses revealed that the mouse PDLC-derived ECM displayed an immature status with active turnover. Moreover, in cultured PDLCs, the gene expression signature is maintained and is similar to that in cells from PDL tissues, with additional characteristics representative of tissue progenitor cells. These observations strongly suggest that PDLC-derived ECM has multiple advantages in periodontal tissue regeneration because it provides an extracellular environment that closely mimics the environment in the native PDL tissue with active ECM turnover, promoting tissue regeneration. Furthermore, cell culture is an indispensable tool for analyzing biological systems in a well-controlled environment. Therefore, understanding the differences between the in vitro and in vivo ECM statuses is crucial. Because our data clearly show the difference between the ECM of cultured PDLCs and that of the native PDL tissue, we can manipulate the ECM of cultured PDLCs to the tissue PDL end by adjusting culture conditions, for example, by supplementing growth factors, applying mechanical force, changing oxygen tension, or using a combination of these. The matrisome profiling that we performed in this study can also be used to evaluate the quality of cultured PDLC-derived ECM for optimal tissue regeneration. Although further studies are required to validate our findings, the multiomics characterization of the PDLC-derived ECM presented in this study provides further insights into developing tissue-specific biomimetic ECM scaffolds for periodontal tissue regeneration.

## Methods

### Ethics statement

The Niigata University Animal Experiment Ethics Committee reviewed and approved the animal experiment protocol (Approved number: SA00532). All animal handling and experiments strictly followed the ARRIVE guidelines for animal research and reporting for in vivo experiments. All methods were performed in accordance with the relevant guidelines and regulations.

### Cell culture

C57BL/6 J mice (male, 5 weeks old, n = 8) were euthanized by carbon dioxide inhalation followed by cervical dislocation. The first and second molars of each quadrant were extracted, and the PDL tissue was dissociated with Liberase DL (Roche, Basel, Switzerland) for 60 min at 37 °C on a horizontal shaker at 100 rpm. The released PDLCs were collected by centrifugation (1000 rpm, 5 min) and maintained in alpha-MEM (Invitrogen, Waltham, MA, USA) containing 10% fetal bovine serum, 1% antibiotics, and antimycotics (Invitrogen). The PDLCs were then seeded onto a 35 mm dish (4 × 10^5^ cells/dish) and cultured for 1 week. The culture medium was supplemented with ascorbic acid (50 µg/mL; Sigma-Aldrich, St. Louis, MO, USA).

### Transcriptome analysis

Total RNA from cultured cells was isolated after seven and 14 days (7D and 14D, respectively, n = 4 each) of culture using the TRIzol reagent (Invitrogen). To isolate total RNA from PDL tissue cells, the molars were harvested as described earlier and treated with TRIzol reagent (n = 14). Next-generation sequencing library was prepared using the MGIEasy RNA Directional Library Prep Set (MGI Tech, Shenzhen, China). Poly(A) mRNA was isolated using the NEBNext Poly(A) mRNA Magnetic Isolation Module (New England Biolabs, Ipswich, MA, USA). Libraries with different indices were multiplexed and loaded onto an MGI DNBSEQ G-400 instrument (MGI Tech) with a 2 × 150-bp paired-end configuration. The sequences were processed and analyzed by Azenta Life Sciences (Tokyo, Japan).

### Laser microdissection

Previously reported proteomic data of the mouse PDL tissue dissected using laser microdissection^5^ were reanalyzed and used in this study. In brief, the mesial and distal sides of the distal root PDL of the upper first molar were dissected from toluidine blue-stained cryosections using an LMD 7000 microdissection system (Leica, Wetzlar, Germany). Approximately 20 cryosections were collected from each tooth (n = 8) for a total of ~ 160 slices to obtain enough protein for proteomic analysis. Each proteomic experiment comprised five biological replicates. Samples were kept at − 80 °C for proteome analysis.

### Proteome analysis

Mouse PDLCs were cultured for 2 weeks in the presence of ascorbic acid (n = 5). The cultures were collected using RIPA buffer (Wako, Osaka, Japan), and the ECM-rich fraction was prepared by centrifugation at 12,000 rpm for 30 min. The precipitate was thoroughly washed with phosphate-buffered saline and double distilled water, lysed with lysis buffer (100 mM Tris–HCl, pH 8.8, 7 M urea, 2% sodium dodecyl sulfate), sonicated, and the protein amount was quantified using the Pierce™ BCA Protein Assay Kit (Thermo Fisher Scientific, Waltham, MA, USA). After reduction with TCEP-HCl (Thermo Fisher Scientific) and alkylation with iodoacetamide (Sigma-Aldrich), tryptic peptides were generated via trypsin digestion.

Trypsin-digested peptides were dissolved in 0.3% formic acid and filtered through a 0.45-μm Ultrafree MC membrane filter (Merck-Millipore, Billerica, MA, USA). Samples containing 0.13 μg peptide were analyzed using direct-injection mode on an Eksigent NanoLC 415 nano-flow liquid chromatography system (Sciex, Framingham, MA, USA) using a 75 µm × 150 mm C18 spray-tip column (3 μm, 120 Å; Nikkyo Technos, Tokyo, Japan) coupled with a TripleTOF 5600 + tandem mass spectrometer (Sciex). Separation involved a 60 min gradient elution using 98% A, 2% B to 68% A, and 32% B at 300 nL/min (A and B refer to mobile phases comprising 0.1% aqueous formic acid and 0.1% formic acid in acetonitrile, respectively). The MS spectrum (250 ms) and the following 10 MS/MS spectra (100 ms each) were acquired in data-dependent mode. The dataset for the mouse PDL tissue obtained using laser microdissection (JPST001418/PXD030430, https://repository.jpostdb.org/entry/JPST001418) was previously published^5^.

Raw data generated using Analysis TF 1.6 (Sciex) were converted to Mascot generic files using the MS Data Converter (Sciex) and searched against a mouse reference protein sequence database (UniProt, Mus *musculus*) using the Mascot search engine (v.2.6; Matrix Science, Boston, MA, USA). The peptide and MS/MS tolerances were set at ± 20 ppm and ± 0.1 Da, respectively. A maximum of one missed cleavage was allowed. The search settings involved cysteine carbamidomethylation as a fixed modification and the following variable modifications: deamidation of asparagine and/or glutamine, N-terminal glutamine to pyroglutamate modification, and oxidation of methionine, proline, and lysine. The target false discovery rate (FDR) was set to < 1%. Proteins detected in at least two out of five samples were considered identified.

### Bioinformatics

For bioinformatics analysis, matrisome genes/proteins were selected from all identified proteins using the Matrisome Annotator (http://matrisomeproject.mit.edu/analytical-tools/matrisome-annotator/)^47^. The occupancy of each matrisome protein was calculated based on the emPAI value^45^ divided by the sum emPAI value of the total matrisome proteins. The matrisome profile was determined as the mean of five biological replicates. Statistical significance was calculated using the Benjamini–Hochberg procedure^48^ with the following thresholds: genes, log (fold change) >|0.2| and log (FDR) > 2; proteins, log (fold change) >|0.08| and log (FDR) > 2. Statistically significant genes/proteins were considered differentially expressed. Overrepresentation analysis was performed using eVITTA v1.3.1^49^ based on the following ontology sources: KEGG Pathway, GO Biological Processes, GO Cellular Components, GO Molecular Functions, Reactome Gene Sets, and WikiPathways. k-Means clustering was conducted using iDEP 1.0^50^. The top 2000 statistically significant genes were clustered (n = 3) based on their expression patterns across all samples and visualized as a heatmap. Overrepresentation analysis was performed in each cluster using eVITTA v1.3.1. The relative abundance of protein was calculated based on the occupancy of each matrisome protein, as described earlier. The occupancy of each protein in PDLCs in culture was divided by that in the PDL tissue. To calculate the relative gene expression, the transcripts per million (TPM) values of RNA-seq data were used. The TPM value for PDLCs in culture was divided by that for the PDL tissue to obtain the relative gene expression.

### Statistical analysis

The results are expressed as the means ± standard deviations (SDs) from four or five independent experiments. Statistical analysis for differences between groups was performed with a two-tailed unpaired *t*-test with Welch’s correction using Prism 9 (GraphPad Software, San Diego, CA, USA), and a *p* value of < 0.05 was considered statistically significant.

### Supplementary Information

Supplementary Information 1.

Supplementary Information 2.

## Data Availability

The datasets generated and/or analyzed during the current study are available in the Japan ProteOme STandard Repository/Database with the data set identifier JPST001418/ PXD030430 (https://repository.jpostdb.org/entry/JPST001418), or from the corresponding author on reasonable request.
