# Supplementary material for: Multiomics analysis of cultured mouse periodontal ligament cell-derived extracellular matrix
Source: Sci Rep. 2024 Jan 3;14:354. doi: 10.1038/s41598-023-51054-8 (PMC10764881; doi:10.1038/s41598-023-51054-8)
Supplement: Supplementary file 1 — Supplementary Information 1. [file 41598_2023_51054_MOESM1_ESM.pdf]

Multiomics analysis of cultured mouse periodontal ligament cell-derived extracellular matrix

Masaru Kaku[1*], Lay Thant[2,3], Azusa Dobashi[1], Yoshiki Ono[1], Megumi Kitami[3], Masaru Mizukoshi[2], Moe Arai[2], Hajime Iwama[2], Kohei Kitami[2], Yoshito Kakihara[3], Masaki Matsumoto[4], Isao Saito[2], and Katsumi Uoshima[1]

[1]Division of Bio-prosthodontics, Faculty of Dentistry & Graduate School of Medical and Dental Sciences, Niigata University, Niigata, Japan
[2]Division of Orthodontics, Faculty of Dentistry & Graduate School of Medical and Dental Sciences, Niigata University, Niigata, Japan
[3]Division of Dental Pharmacology, Faculty of Dentistry & Graduate School of Medical and Dental Sciences, Niigata University, Niigata, Japan
4Department of Omics and Systems Biology, Graduate School of Medical and Dental Sciences, Niigata University, Niigata, Japan
[*]Correspondence: Masaru Kaku; Email: kakum@dent.niigata-u.ac.jp

	Cell	Tissue	Fold Change	p
**Collagens**	43.8%	87.3%	0.5	4.E-07
**Proteoglycans**	12.4%	2.3%	5.3	8.E-08
**ECM Glycoproteins**	25.1%	3.6%	7.0	5.E-06
**ECM Regulators**	6.0%	1.7%	3.5	1.E-04
**ECM-affiliated Proteins**	9.7%	4.5%	2.2	4.E-03
**Secreted Factors**	3.1%	0.6%	5.5	1.E-03

Supplemental Fig. S1. Proportional change in each matrisome subclass in cultured mouse periodontal ligament cells and PDL tissues.

Collagens

GeneSymbol	Cell Ave.	Tissue Ave.	Cell/Tissue	p
Col1a1	20.68965%	58.51006%	0.4	2.E-07
Col1a2	10.46891%	23.00455%	0.5	2.E-05
Col12a1	4.75262%	0.51486%	9.2	4.E-06
Col6a1	2.08512%	0.08658%	24.1	4.E-06
Col6a2	1.64527%	0.07605%	21.6	6.E-05
Col5a2	1.45391%	0.18530%	7.8	2.E-04
Col3a1	1.16465%	4.65966%	0.2	5.E-06
Col5a1	0.53218%	0.07743%	6.9	5.E-04
Col11a1	0.45205%	0.06059%	7.5	6.E-05
Col2a1	0.11142%	0.07772%	1.4	1.E-02
Col8a1	0.10179%	0%		
Col18a1	0.08698%	0%		
Col14a1	0.04988%	0%		
Col4a2	0.04962%	0%		
Col23a1	0.04189%	0%		
Col28a1	0.02791%	0.02509%	1.1	8.E-01
Col15a1	0.01957%	0%		
Col7a1	0.01869%	0.00444%	4.2	1.E-04
Col11a2	0.01492%	0.01404%	1.1	9.E-01
Col8a2	0.01408%	0%		
Col4a1	0.01284%	0.00120%	10.7	1.E-01
Col16a1	0.01138%	0.02134%	0.5	1.E-01
Col4a3	0.00890%	0.00876%	1.0	1.E+00
Col24a1	0.00476%	0.00633%	0.8	7.E-01
Col27a1	0%	0.00240%		

Proteoglycans

GeneSymbol	Cell Ave.	Tissue Ave.	Cell/Tissue	p
Aspn	2.93914%	0.83785%	3.5	7.E-05
Lum	2.06063%	0.66485%	3.1	5.E-05
Bgn	1.99820%	0.35071%	5.7	3.E-05
Prelp	1.99024%	0.01154%	172.5	1.E-05
Ogn	1.27423%	0.15522%	8.2	8.E-05
Dcn	0.92544%	0.21019%	4.4	6.E-06
Fmod	0.82539%	0.06585%	12.5	9.E-05
Hspg2	0.32263%	0.00630%	51.2	1.E-05
Vcan	0.02056%	0%		
Omd	0%	0.00760%		
Podn	0%	0.00464%		

ECM Glycoproteins

GeneSymbol	Cell Ave.	Tissue Ave.	Cell/Tissue	p
Postn	12.47216%	2.37041%	5.3	2.E-05
Tnn	1.34249%	0.31322%	4.3	2.E-04
Fn1	1.13647%	0.00581%	195.7	7.E-06
Thbs1	0.94238%	0.00805%	117.1	3.E-04
Fbln2	0.87166%	0%		
Mfap5	0.80773%	0%		
Srpx2	0.67091%	0.01125%	59.6	3.E-03
Efemp1	0.65840%	0%		2.E-03
Emilin1	0.64026%	0.02353%	27.2	1.E-05
Tnc	0.53812%	0.00302%	178.4	4.E-04
Efemp2	0.53128%	0%		4.E-04
Thbs2	0.50326%	0.00160%	314.8	5.E-04
Matn4	0.45779%	0%		
Srpx	0.41564%	0.01502%	27.7	8.E-05
Mfap2	0.34609%	0%		
Dpt	0.34491%	0%		
Aebp1	0.32785%	0.08801%	3.7	9.E-03
Fbln5	0.31887%	0.00396%	80.6	4.E-03
Fbn1	0.28454%	0.00961%	29.6	6.E-02
Mfap4	0.17952%	0%		
Tgfbi	0.17578%	0%		
Cthrc1	0.14742%	0%		
Nid1	0.13030%	0.00392%	33.2	8.E-04
Nid2	0.12480%	0%		
Ltbp2	0.10317%	0%		
Tinagl1	0.08612%	0%		
Igsf10	0.08508%	0%		
Fbln1	0.06624%	0%		
Pcolce	0.05123%	0.06149%	0.8	6.E-01
Lamc1	0.04791%	0.00262%	18.3	1.E-02
Ecm1	0.03802%	0%		
Fbn2	0.03166%	0%		
Lamb1	0.02396%	0%		
Ltbp1	0.02357%	0%		
Fgg	0.02285%	0.08010%	0.3	4.E-02
Fgb	0.02131%	0.04894%	0.4	1.E-01
Emilin2	0.01975%	0%		
Lama4	0.01740%	0%		
Lamb2	0.01653%	0%		
Thsd4	0.01404%	0%		
Eln	0.01328%	0%		
Matn2	0.01176%	0%		
Agrn	0.00702%	0.00170%	4.1	5.E-04
Lama5	0.00625%	0%		
Ltbp3	0.00367%	0%		
Hmcn2	0.00122%	0%		
Mgp	0%	0.27083%		
Fga	0%	0.09179%		
Spp1	0%	0.03770%		
Thbs4	0%	0.03617%		
Ibsp	0%	0.01960%		
Vwa5a	0%	0.01633%		
Sparc	0%	0.01610%		
Thbs3	0%	0.01016%		
Adipoq	0%	0.00969%		
Vtn	0%	0.00403%		
Ecm2	0%	0.00293%		
Sned1	0%	0.00171%		

Supplemental Fig. S2. Abundance of core matrisome proteins in cultured mouse PDLCs and mouse PDL tissue ECM. Occupancy was calculated based on the emPAI value of each matrisome protein divided by the sum emPAI value of total matrisome proteins. The protein order in each matrisome subclass corresponds to the descending order of emPAI value in the Cell ECM.

ECM Regulators

GeneSymbol	Cell Ave.	Tissue Ave.	Cell/Tissue	p
Serpinh1	2.58680%	0.83572%	3.1	1.E-03
Htra1	1.67478%	0%		
Htra3	0.44343%	0%		
Loxl1	0.35049%	0%		
Tgm2	0.24372%	0%		
Lox	0.23590%	0%		
Adamtsl4	0.20236%	0%		
Loxl2	0.07224%	0%		
Ctsb	0.03355%	0%		
Itih2	0.03001%	0%		
F13a1	0.02106%	0%		
Mmp2	0.02063%	0%		
A2m	0.00398%	0%		
Serpinf1	0%	0.22856%		
Serpina3k	0%	0.12148%		
Serpina1b	0%	0.08902%		
Serpina1d	0%	0.08880%		
Serpina1a	0%	0.07135%		
Serpina1e	0%	0.04285%		
Kng1	0%	0.02675%		
Pzp	0%	0.02523%		
F2	0%	0.02132%		
Hrg	0%	0.01611%		
F10	0%	0.01559%		
Plg	0%	0.01476%		
Serpinc1	0%	0.01433%		
Serping1	0%	0.01179%		
Serpine1	0%	0.00629%		
Mmp11	0%	0.00548%		
F9	0%	0.00459%		
Egln2	0%	0.00416%		
Itih1	0%	0.00360%		

ECM-affiliated Proteins

GeneSymbol	Cell Ave.	Tissue Ave.	Cell/Tissue	p
Lgals1	5.72782%	0.41795%	13.7	5.E-03
Anxa2	1.62457%	1.59606%	1.0	1.E+00
Clec11a	0.57062%	0.03014%	18.9	1.E-06
Anxa1	0.48862%	0.54926%	0.9	7.E-01
Lgals3	0.42165%	0.02957%	14.3	1.E-03
Anxa5	0.25809%	1.32116%	0.2	4.E-03
C1qtnf3	0.23867%	0%		
Lgals9	0.13212%	0%		
Anxa6	0.08911%	0.23774%	0.4	2.E-02
Grem1	0.06441%	0.00000%		
Sftpd	0.03349%	0.01134%	3.0	6.E-02
Plxna1	0.00249%	0%		
Hpx	0%	0.10477%		
Lman1	0%	0.05099%		
Anxa4	0%	0.04950%		
Anxa7	0%	0.02820%		
Anxa11	0%	0.02127%		
Colec12	0%	0.00874%		

Secreted Factors

GeneSymbol	Cell Ave.	Tissue Ave.	Cell/Tissue	p
S100a4	1.65364%	0.06988%	23.7	7.E-03
S100a6	0.89656%	0.18545%	4.8	1.E-05
S100a13	0.18252%	0%		
S100a11	0.17901%	0.16405%	1.1	6.E-01
S100a10	0.06801%	0.04528%	1.5	6.E-01
Angptl2	0.03159%	0%		
Chrdl1	0.02500%	0%		
Wnt10a	0.01587%	0%		
Pdgfd	0.01347%	0%		
Megf6	0.00577%	0%		
Clcf1	0%	0.02958%		
S100a9	0%	0.01882%		
Angptl4	0%	0.01146%		
Il11	0%	0.00959%		
Wfikkn2	0%	0.00452%		
Ccbe1	0%	0.00440%		
Scube3	0%	0.00272%		

Supplemental Fig. S3. Abundance of matrisome-associated proteins in cultured mouse PDLCs and mouse PDL tissue ECM. Occupancy was calculated based on the emPAI value of each matrisome protein divided by the sum emPAI value of total matrisome proteins. Protein order in each matrisome subclass corresponds to the descending order of emPAI value in the Cell ECM.

Supplemental Fig. S4. Kaku et al.

(a)

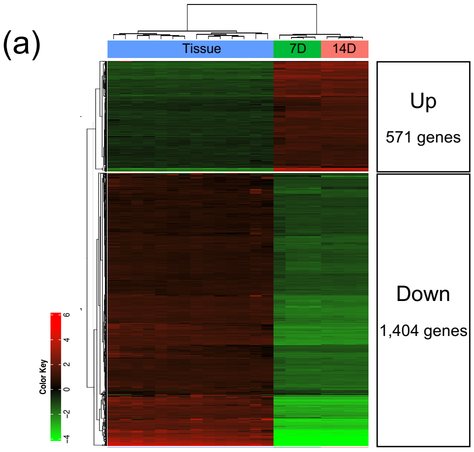

(b) **GO Biological Process**

	FDR	Fold. enriched	Pathway
**Up**	2.63E-14	1.95	Anatomical structure morphogenesis
	1.13E-13	4.69	Extracellular matrix organization
**Down**	1.37E-25	7.49	Keratinization
	5.44E-17	4.11	Keratinocyte differentiation

(c) **GO Cellular component**

	FDR	Fold. enriched	Pathway
**Up**	1.99E-14	2.09	Extracellular region
	1.99E-14	3.61	External encapsulating structure
**Down**	1.48E-39	4.43	Ribosome
	1.48E-39	4.76	Ribosomal subunit

(d) **GO Molecular Function**

	FDR	Fold. enriched	Pathway
**Up**	7.52E-11	6.04	Extracellular matrix structural constituent
	9.82E-06	3.90	Glycosaminoglycan binding
**Down**	3.28E-43	5.54	Structural constituent of ribosome
	1.29E-39	3.17	Structural molecule activity

Supplemental Fig. S4. Gene expression profiling of cultured mouse periodontal ligament cells (PDLCs) and mouse PDL tissues. (a) Heatmap showing k-means clustering of the top 2000 differentially expressed genes (DEGs). Expression values of each biological replicate are indicated according to a color gradient. The enriched gene ontology (GO) Biological Process (b), GO Cellular Component (c) and GO Molecular Function (d) of 571 upregulated and 1404 downregulated genes in cultured PDLCs. ECM-related enriched terms are shown in red. FDR, false discovery rate.

Gene expression profile of collagen-modifying enzymes

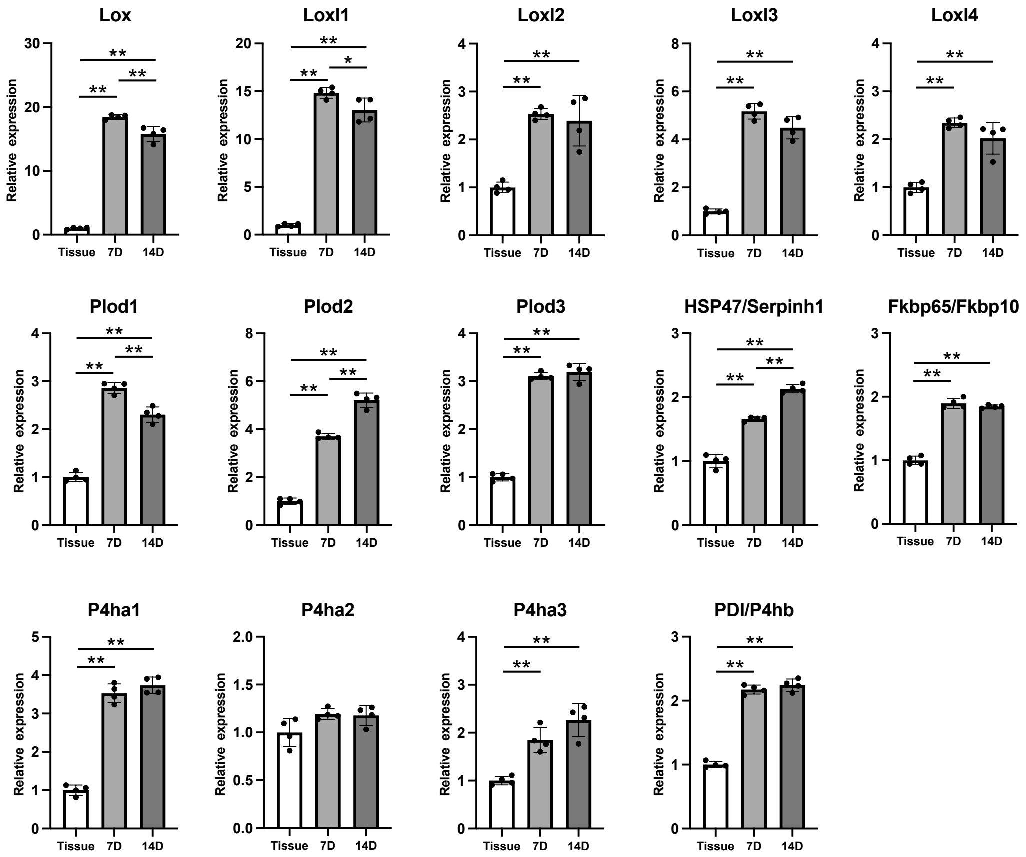

Supplemental Fig. S5. Gene expression profile of collagen-modifying enzymes in cultured mouse PDLCs and mouse PDL tissue. Relative gene expression of collagen-modifying enzymes in cultured PDLCs compared with that in cells from PDL tissues. 77D, cultured PDLCs on day 7; 14D, cultured PDLCs on day 14. *, p<0.05; **, p<0.01.
